# Expression of FABP4, adipsin and adiponectin in Paneth cells is modulated by gut *Lactobacillus*

**DOI:** 10.1038/srep18588

**Published:** 2015-12-21

**Authors:** Xiaomin Su, Hui Yan, Yugang Huang, Huan Yun, Benhua Zeng, Enlin Wang, Yu Liu, Yuan Zhang, Feifei Liu, Yongzhe Che, Zhiqian Zhang, Rongcun Yang

**Affiliations:** 1 Department of Immunology, Nankai University School of Medicine, Tianjin, P. R. China; 2 State Key Laboratory of Medicinal Chemical Biology, Tianjin, P. R. China; 3 Key Laboratory of Bioactive Materials Ministry of Education, Nankai University, Tianjin, P. R. China; 4 The Fourth Military Medical University, Chongqing, P. R. China

## Abstract

We here found that intestinal epithelial Paneth cells secrete FABP4, adipsin and adiponectin in both mice and human. Deletion of Paneth cell results in the decrease of FABP4, adipsin and adiponectin not only in intestinal crypt cells but also in sera, suggesting that they may influence the state of the whole body. We also demonstrate that expression of FABP4, adipsin and adiponectin may be modulated by specific gut microbiota. In germ-free (GF) mice, the expression of FABP4, adipsin and adiponectin were lower or difficult to be detected. Feces transplantation promoted the expression of FABP4, adipsin and adiponectin in gut epithelial Paneth cells. We have found that *Lactobacillu*s NK6 colony, which has the highest similarity with *Lactobacillus taiwanensis strain* BCRC 17755, may induce the expression of FABP4, adipsin and adiponectin through TRAF2 and TRAF6 ubiquitination mediated NF-κB signaling. Taken together, our findings set up a novel mechanism for FABP4, adipsin and adiponectin through gut microbiota mediating expression in gut Paneth cells.

Metabolism associated factors such as the fatty acid binding protein 4 (FABP4), adipsin and adiponectin play a significant role in the pathogenesis of a cluster of metabolic syndromes such as hypertriglyceridemia, insulin resistance and atherosclerosis^1^,^2^,^3^,^4^. Adipose tissues^4^ and adipose associated macrophages^5^ can secrete these factors to affect the occurrence and development of metabolic diseases. However, it is difficult to explain some observations such that metabolic syndrome can occur in lean individuals^6^,^7^ and that some morbidly obese ones are metabolically healthy^8^,^9^. These facts imply that adipose tissues may not be a primary origin of metabolic syndrome. Previous studies have suggested that gut epithelial Paneth cells, which are located at the base of the crypts of Lieberkühn, possess the potential to express metabolism associated factors such as adiponectin and adipsin (complement factors D)^10^,^11^. We here demonstrate that intestinal epithelial Paneth cells secrete FABP4, adipsin and adiponectin in both mice and human. We also found that the expression of FABP4, adipsin and adiponectin may be regulated by gut bacteria *Lactobacillu*s NK6 colony. Thus, our findings set up a novel regulating axis for FABP4, adipsin and adiponectin through gut microbiota mediating expression in gut Paneth cells.

## Results

### Expression of FABP4, adipsin and adiponectin in intestinal Paneth cells

**Figure 1:**
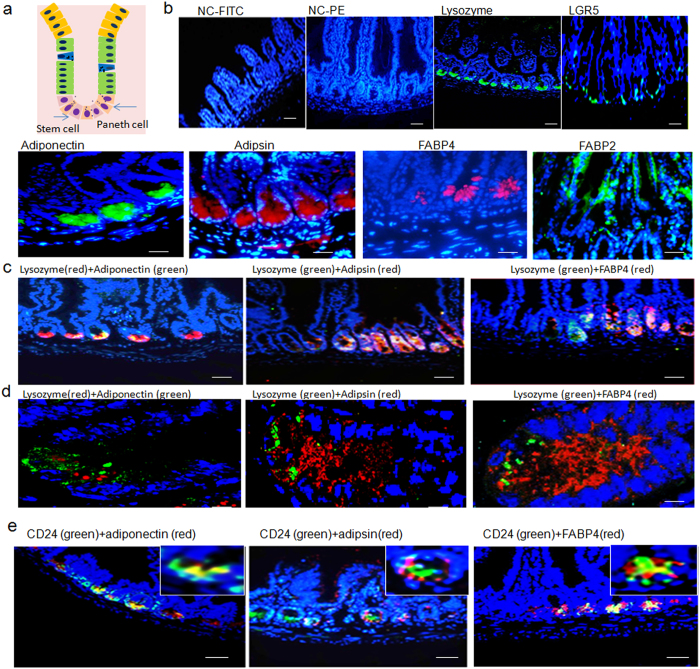
Expression of FABP4, adipsin and adiponectin in intestinal Paneth cells. (**a**) Schematic representation (right) and staining (left) of the Paneth cells and putative intestinal stem cells. Paneth cells (lysozyme positive) and putative intestinal stem cells (LGR5 positive) localized at the crypt base. Intestinal tissues were sliced and stained using pooled isotypic control (NC) with FITC-labeled (NC-FITC) or PE-labeled (NC-PE) secondary antibodies, anti-lysozyme (lysozyme) and anti-LGR5 (LGR5) antibodies followed by fluorescence-labeled second antibodies. Green indicated lysozyme or LGR5 positive. Nuclei were stained by DAPI (blue). (**b**) Immunostaining of FABP4, adipsin, adiponectin (adipon) and FABP2 in ileum section from *WT* mice. Red and green indicated respectively FABP4, adipsin or adiponectin (adipon) and FABP2. Scale bar, 20μm. (**c**) Double immunostaining of FABP4, adipsin and adiponectin (adipon) with lysozyme in ileum section from *WT* mice. Scale bar, 40μm. (**d**) Analysis of confocal microscope of FABP4, adipsin and adiponectin (adipon) with lysozyme in ileum section from *WT* mice after double staining. Scale bar, 5 μm. (**e**) Double immuno-staining of adipsin, adiponectin and FABP4 with CD24 in the colon fragments of mice. Colon tissues were sliced and stained using anti-adipsin, anti-adiponectin and anti-FABP4 with anti-CD24 antibodies followed by fluorescence-labeled second antibodies. Green indicated CD24; Red indicated adipsin, adiponectin or FABP4. Nuclei were stained by DAPI (blue). Scale bar, 40 μm.

**Figure 2:**
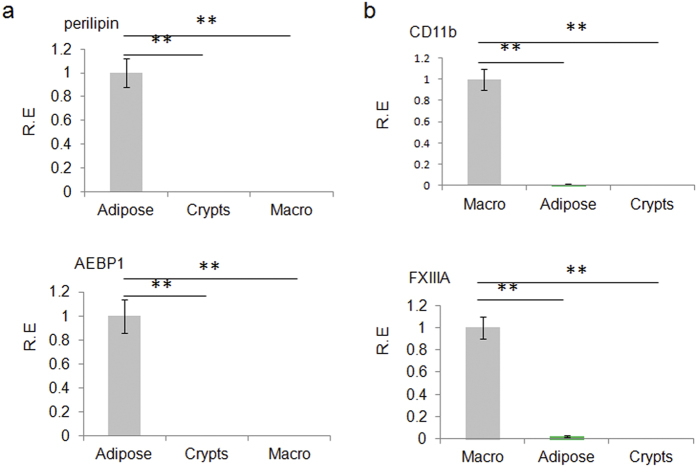
Expression of perilipin, AEBP-1, FXIIIA and CD11b in intestinal crypt cells, adipose tissues and macrophages. (**a**) qRT-PCR of perilipin and AEBP-1 in intestinal crypt cells (Crypts), adipose tissues (Adipose) and macrophages (Macro). (**b**) qRT-PCR of FXIIIA and CD11b in intestinal crypt cells (Crypts), adipose tissues (Adipose tissues) and macrophages (Macro). Intestinal crypt cells were isolated according to the protocol described in the material and methods. Adipose tissues were from mouse fat pat. Macrophages were harvested by peritoneal lavage after 3–4 days following an i.p. injection of 4% sterile thioglycolate medium and then cultured in six-well plates and incubated at 37 °C and 5% CO_2_. After 6 h, the cells were washed to separate out any nonadhesive cells. R. E., relative expression. *P < 0.05 and **P < 0.01 (t-test, mean ± SD). The data were representative of three independent experiments.

Previous studies have implied that gut epithelial Paneth cells may potentially express metabolism associated factors such as adiponectin and adipsin (complement factors D)^10^,^11^. Our immunohistochemistry and immunoblot results validated these implications and detected their expression at the similar position in gut tissues (Fig. 1a–d and Supplementary Fig. S1). Meanwhile, at the base of the crypts we also found the expression of FABP4, which was different from FABP2 widely located in gut epithelial tissues (Fig. 1a–d and Supplementary Fig. S1). These metabolism associated factors were also detected in the crypts of jejunum, ileum and colon tissues (Fig. 1e and Supplementary Fig. S2). We also analyzed the perilipin (a specific marker of adipose cells), AEBP1 (adipocyte enhancer-binding protein 1, a specific marker of preadipose cells) and CD11b and FXIIIA (macrophages specific marker)^12^,^13^,^14^ in the isolated crypts cells. No perilipi, AEBP1, CD11b and FXIIIA were detected, indicating that the isolated crypts cells are not contaminated by adipose cells, preadipose cells and macrophages (Fig. 2). Notably, mouse intestinal epithelial cells (MIEC) from mouse embryonic intestinal cells, could also express FABP4, adipsin and adiponectin (Supplementary Fig. S3), implying that Paneth cells were not the only source for FABP4 and adipsin in gut epithelial tissues.

### Deletion of Paneth cells impairs the expression of FABP4, adipsin and adiponectin in gut crypt cells

**Figure 3:**
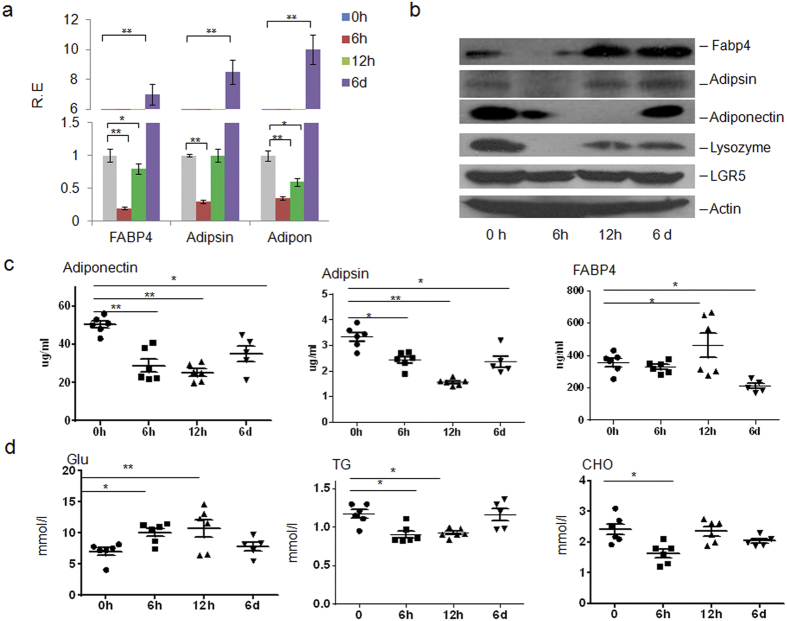
Deletion of Paneth cells impairs the expression of FABP4, adipsin and adiponectin in gut crypt cells. (**a,b**) qRT-PCR (**a**) and immunoblot analyses (**b**) of intestinal crypts with and without dithizone treatments. The mice were intravenously injected by dithizone and killed at the indicated time (male, n = 6 or indicated number). R. E., relative expression. (**c**) Serum levels of FABP4, adipsin and adiponectin in mice with (6h, 12h and 6 days after dithizone) and without (0h) dithizone treatments. (**d**) Serum levels of glucose (Glu), triglyceride (TG) and cholesterol (CHO) in fasting mice with (6h, 12h and 6 days after dithizone) and without (0h) dithizone treatments. *P < 0.05 and **P < 0.01 (*t*-test, mean ± SD in a; Mann-Whitney U test in c,d). The data were a representative of three independent experiments.

To exactly determine the importance of Paneth cells to produce FABP4, adipsin and adiponectin, we employed zinc depletion experiment with dithizone, which may deplete the secretary granules in Paneth cells^15^ and damages Paneth cells^15^,^16^,^17^. Consistent with other results^15^, dithizone treatment eliminated lysozyme staining in Paneth cells, whereas lysozyme staining was still strong in crypt cells of mice treated with control Li2CO3 (Fig. 3a,b and Supplementary Fig. S4). Importantly, this treatment led to the profound decrease of FABP4, adipsin and adiponectin in the isolated crypts (Fig. 3a,b and Supplementary Fig. S4), suggesting that Paneth cells were indeed an important source of these factors. A significant reduction was also detected in serum adipsin and adiponectin (Fig. 3c). Notably, serum levels of triglyceride (TG), glucose (Glu) and cholesterol (Cho) were also profoundly affected by Paneth cell depletion (Fig. 3d). These changes in serum have a great significance because it suggests that the action range of these Paneth cell derived factors are not restricted to intestine, but may be extended to the serum and further, the whole body, which made them able to regulate the systematic metabolism and account for some related diseases. Since metabolism-associated factors such as adiponectin may be considered as negative acute phase protein, we also measured inflammation parameters such as TNF α and IL-6. However, their levels were rather low in sera of both treated and untreated mice (unshown). This may be absence of inflammation in these treated and untreated mice. However, our results clearly show that gut epithelial Paneth cells may be another source of adipsin, adiponectin and FABP4 in addition to adipose tissues, which could largely influence the metabolism of the whole body.

### Expression of FABP4, adipsin and adiponectin is modulated by gut microbiota *Lacbacillus*

**Figure 4:**
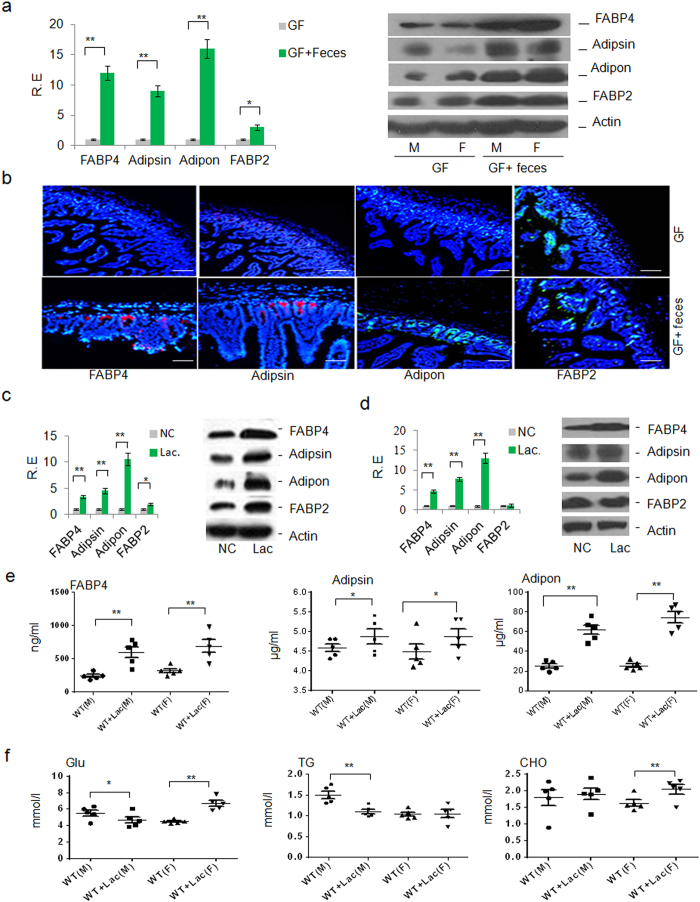
Expression of FABP4, adipsin and adiponectin is modulated by gut microbiota *Lacbacillus*. (**a**) qRT-PCR and immunoblot analyses of epithelial crypts in ileum fragments of germ-free (GF) mice (n = 10 (male, 5; female, 5)) and feces transplanted GF mice (GF + feces, n = 10 (male (M), 5; female (F), 5)). Actin, a loading control, was detected by anti-β-actin antibody. R. E., relative expression. (**b**) Immuno-staining of ileum fragments of germ-free (GF) mice and GF mice transplanted with *WT* mouse feces (GF + feces). Red and green indicated respectively FABP4, adipsin or adiponectin (adipon) and FABP2. Scale bar, 40 μm. The result is a representative of 6 samples. (**c**) RT-PCR and immunoblot analyses of ileum epithelial crypts in mice after *in vivo* transplantation with (Lac, N = 10 (male, 5; female, 5)) or without (NC, N = 10 (male, 5; female, 5)) *Lacbacillus* NK6. Actin, a loading control. R. E., relative expression. (**d**) qRT-PCR and immunoblot analyses of ileum epithelial crypts after *in vitro* stimulation with (Lac) or without (NC) *Lacbacillus* NK6. Actin, a loading control. (**e**) Serum levels of FABP4, adipsin and adiponectin (adipon) after transplantation with (*WT* + Lac, N = 10 (male (M), 5; female (**F**), 5)) or without (*WT*, N = 10 (male (M), 5; female (F), 5)) *Lacbacillus* NK6. (**f**) Serum levels of glucose (Glu), triglyceride (TG) and cholesterol (CHO) in fasting mice after transplantation with (*WT* + Lac, N = 10 (male (M), 5; female(F), 5)) or without (*WT*, N = 10 (male(M), 5; female(F), 5)) *Lacbacillus* NK6. *P < 0.05 and **P < 0.01 (*t*-test, mean ± SD in a, c, d; Mann-Whitney U test in e, f). The data were representative of three independent experiments.

In addition to this finding that gut Paneth cells have an influence on these metabolism associated factors, another important phenomenon is that almost all the metabolic syndromes have a close relationship with the altered gut microbiota. Since both of them could control metabolism in the whole body, it is intriguing for possible regulation of gut microbiota in the expression of FABP4, adipsin and adiponectin in Paneth cells. Paneth cells, which are highly specialized epithelial cells^18^, directly sense gut microbiota and maintain homeostasis at the intestinal host-microbial interface^19^. To determine the effects of gut microbiota on the expression of FABP4, adipsin and adiponectin in gut epithelial Paneth cells, we first employed the germ-free (GF) mice, which are often used as a model to explore the effect of gut microbiota on the metabolic diseases. The expression of FABP4, adipsin and adiponectin in GF mice was low (Fig. 4a,b). However, wild-type (*WT*) mouse feces transplantation could reverse and further promote their expression in gut epithelial Paneth cells (Fig. 4a,b). In addition, the changed expression of FABP4, adiposin and adiponectin also occurred in leptin deficient (Ob/Ob) mice (Supplementary Fig. S5a, b), which have a higher proportion of Firmicutes to Bacteroidetes than lean mice, in high-fat diet mice (Supplementary Fig. S5c, d), which have a different gut microbiota composition and also in pan-antibiotics treated mice (Supplementary Fig. S5e, f). Notably, the expression of FABP4, adipsin and adiponectin was also upregulated by vitamin A and its metabolites 9-cis-retinoid acid (9-cis) and all-trans retinoid acid (ATRA) in gut epithelial crypt cells (Supplementary Fig. S6), in which vitamin A metabolism may be regulated by gut microbiota. All of these evidences support the conclusion that the ability of Paneth cells to express these factors may be modulated by gut microbiota. Thus, it could be seen that gut microbiota influence systemic metabolism at least in part by regulating the expression of FABP4, adipsin and adiponectin in Paneth cells.

Several reports have narrowed the range of bacteria associated with metabolic syndrome down to a few kinds of strains. For example, *Lactobacillus spp* plays a key role in the physiopathology of obesity, type 2 diabetes, and metabolic inflammation^20^; whereas *Lactobacillus rhamnosus* and *Lactobacillus plantarum* were reported to be able to reduce the fat content of mouse adipose tissues^21^. All of these imply that gut *Lactobacillus* might be involved in the regulation of metabolism. Indeed, we found that *Lactobacillus* NK6, which was isolated from *WT* mice and has the highest similarity with *Lactobacillus taiwanensis* strain BCRC 17755 (Supplementary Fig. S7–8), could mediate the expression of FABP4, adipsin and adiponectin (Fig. 4c)*. In vivo* transplantation of this strain could affect the levels of FABP4, adipsin and adiponectin in both intestinal tissues (Fig. 4d) and sera (Fig. 4e). Furthermore, the serum levels of TG, Glu and Cho in the transplanted mice also exhibited remarkable changes, corresponding to the whole body effects of these factors, although there exist differences between male and female mice (Fig. 4f). However, as a comparison, expression of FABP4, adipsin and adiponectin were not changed in adipose tissues and macrophages of mice with or without *Lactobacillus* treatment (Supplementary Fig. S9), indicating that these changes only correlate with the metabolism associated factors secreted by gut Paneth cells. Taken together, our results suggested that gut *Lactobacillus* NK6 may affect the systematic metabolism through affecting FABP4, adipsin and adiponectin expression in gut epithelial Paneth cells.

### *Lacbacillus*-mediated FABP4, adiposin and adiponectin is through TRAF2/6 ubiquitination mediated NF-κB pathway

**Figure 5:**
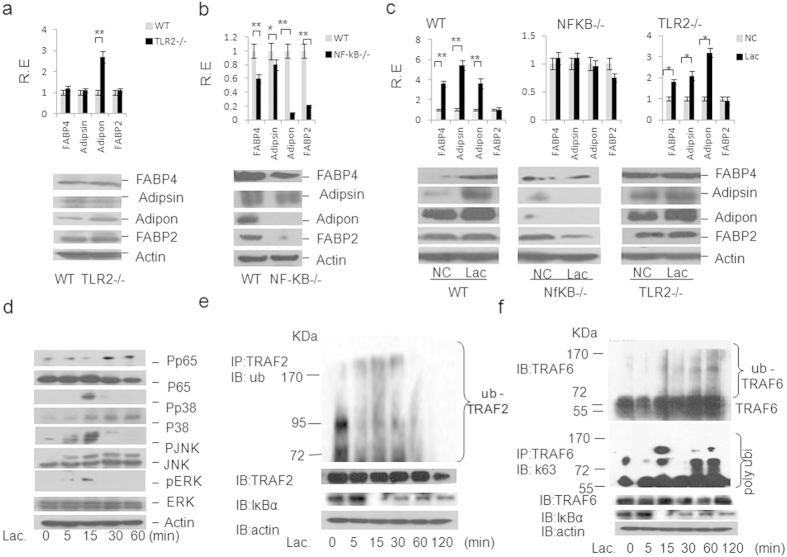
*Lacbacillus*-mediated FABP4, adiposin and adiponectin is through TRAF2/6 ubiquitination mediated NF-κB pathway. (**a**) qRT-PCR (upper) and immunoblot (lower) of FABP4, adipsin, adiponectin and FABP2 in the jejunum epithelial crypt cells of *WT* and TLR2 deficient (−/−) mice (n = 6). R. E., relative expression. (**b**) qRT-PCR (upper) and immunoblot (lower) of FABP4, adipsin, adiponectin and FABP2 in the ileum epithelial crypt cells of *WT* and NF-κB deficient (−/−) mice (n = 6). R. E., relative expression. (**c**) qRT-PCR and immunoblot of FABP4, adipsin, adiponectin and FABP2 in the ileum epithelial crypts of *WT*, TLR2 and NF-κB deficient (−/−) mice with (Lac) or without (NC) *Lacbacillus* NK6 stimulation. R. E., relative expression. (**d**) Phosphorylation analyses of p65, p38, JNK, and ERK in the intestinal crypts after exposed to *Lacbacillus* NK6. Murine intestinal crypts were stimulated with *Lacbacillus* NK6 and lysed at the indicated time. Phosphor-p65, p38, -ERK and -JNK were detected using anti-phosphor-p-65, p38, -ERK or –JNK antibodies. Actin, a loading control, was detected by anti-β-actin antibody. (**e**) Analyses of TRAF2 ubiquitination in intestinal crypt cells after exposed to *Lacbacillus* NK6. Immunoblot analysis of ubiqitinated TRAF2 and TRAF2 immunoprecipitated from intestinal crypt cells stimulated with *Lacbacillus* NK6 at the indicated time (upper blots), and TRAF2, IκBα and actin (below blot) in the same cells without immunoprecipitation. IP, immunoprecipitation; IB, immunoblot assay. (**f**) Analyses of TRAF6 ubiquitination in intestinal crypt cells after exposed to *Lacbacillus* NK6. Immunoblot analysis of K63-linked ubiquitination (K63-Ub) (middle blot) of endogenouse TRAF6 immunoprecipitated from intestinal crypts stimulated with *Lacbacillus* NK6, and immunoblot analysis of Ub-TRAF6 (top blot), TRAF6, IkBα and actin (below blots) in the same cells without immunoprecipitation. PolyUbi., polyubiquitination; IP, immunoprecipitation; IB, immunoblot assay. *P < 0.05 and **P < 0.01 (*t*-test in a, b, c, mean ± SD). The data were at least a representative of three independent experiments.

*Lactobacillus,* as a gram positive bacterium, was also potentially recognized by TLR2 receptor. But beyond our expectation, the level of FABP4, adipsin and adiponectin in the gut epithelial crypts of TLR2 deficient mice was not lower than those in control mice (Fig. 5a, Supplementary Fig. S10). However, NF-κB deficient mice exhibited a remarkably reduced level of FABP4, adipsin and adiponectin in their intestinal epithelial crypt cells (Fig. 5b, Supplementary Fig. S10–11). *In vitro* experiments also showed that intestinal crypt cells from NF-κB deficient mice had a lower level of FABP4, adipsin and adiponectin as compared to those of *WT* mice after exposed to heat-inactivated *Lactobacillus* (Fig. 5c). This reflected that in intestinal crypt cells, the regulation of *Lactobacillus* on the expression of metabolism associated factors was largely mediated by the downstream signaling molecule NF-κB, but not initiated by TLR2. However, this malfunction of TLR2 was not general, because there were difference in the expression of FABP4, adipsin and adiponectin among jejunum, ileum and colon fragments of both TLR2 and NF-κB deficient mice (Fig. 5c and Supplementary Fig. S11). Further studies showed that isolated *Lactobacillus* NK6 could extensively activate the gut cell signal pathways, including NF-κB and MAPK pathways in intestinal crypts (Fig. 5d). TRAF2 and TRAF6 in gut epithelial crypt cells were ubiquitinated, especially TRAF6 K63 ubiquitination after exposed to *Lactobacillus* NK6 (Fig. 5e,f). Thus, gut microbiota *Lactobacillus* NK6 triggered FABP4, adipsin and adiponectin is through TRAF2 and TRAF6 ubiquitination mediated NF-κB signaling pathways. In human, some clinical evidences have implied the effects of adipsin, adiponectin and FABP4 from gut epithelial Paneth cells on colitis associated metabolic syndrome. Statistically, the number of Paneth cells were found dramatically increased in the caecum, ascending, transverse and descending colons in patients with ulcerative colitis (UC) and Crohn’s disease (CD)^22^. Our results further exhibited a stronger staining of lysozyme and LGR-5 in the gut epithelial crypt of patients with colitis (Supplementary Fig. S12a) too. Meanwhile, stronger staining of FABP4, adipsin and adiponectin in the epithelial crypts of ulcerative colitis patients could be detected (Supplementary Fig. S12b). Thus, increased FABP4, adipsin and adiponectin in colitic epithelial Paneth cells may be involved in the occurrence and development of metabolic syndrome.

## Discussion

Paneth cells may produce FABP4, adipsin and adiponectin, which can regulate multiple physiological functions and are implicated in the pathogenesis of clinical entities, such as the metabolic syndrome in autocrine, paracrine and endocrine manners. Therefore, these cells may be considered as a modulator of systemic metabolism. The regulation of FABP4, adipsin and adiponectin in gut epithelial Paneth cells by gut microbiota *Lactobacillus* may offer a clue to disclose the association between gut microbiota and metabolic syndrome. Our studies suggest a new mechanism for systemic metabolism from gut microbiota, FABP4, adipsin and adiponectin in gut epithelial Paneth cells to metabolic syndrome. Because of the less bias of intestinal crypt cells distribution than that of adipose tissues between obese and lean people, our findings explain the disjunction between metabolism malfunction and obesity level in some cases. Finally, the observed abnormal state of Paneth cells and the metabolism associated factors in some metabolism related diseases also stimulates an important consideration regarding therapeutic strategies for these diseases.

## Methods

### Reagents

Anti-mouse FABP4 (ab13979, Abcam), adipsin (M-120, Santa cruz), adiponectin (19F1, Abcam), FABP2 (9A9B7B3, Milipore), LGR5 (EPR3065Y, Abcam), lysozyme (EPR2994, Abcam), CD24 (Mi/69, Abcom), lysozyme (W-20, Santa), p38(D13E1,Cell Signaling), JNK(sc-137020, Santa), ERK(197G2,Cell Signaling), STAT3 (124H6, Cell Signaling), phosphorylated-STAT3 (EP2147Y,Abcam), phosphorylated-p38 (pp38) (D3F9,Cell Signaling), phosphorylated -JNK (pJNK)(sc-81502,Santa), phosphorylated- ERK (pERK) (D13.14.4E,Cell Signaling), phosphorylated -IκBα (pIkBα) (14D4,Cell Signaling), IκBα (L35A5,Cell Signaling), p-65 (sc8008, Santa),phosphorylated-p-65 (sc-52401,Santa), ubiquitin (YT4793,Immunoway), K63-Ub (HWA4C4,Enzo), K48-Ub (D9D5,Cell Signaling), TRAF2 (EPR6048, Epitomics), TRAF6 (sc-8408,Santa), β-actin (sc-47778,Santa) antibodies, IFKine green conjugated anti-goat IgG (Abbkine), IFKine red conjugated anti-rabbit IgG (Abbkine),

IFKine red conjugated anti-mouse IgG (Abbkine), fluorescein-conjugated anti-mouse IgG (ZSGB-BIO), fluorescein-conjugated anti-rat IgG (ZSGB-BIO), rhodamine-conjugated anti-rabbit IgG (ZSGB-BIO), and DAPI staining reagent (D9542,Sigma) were purchased. Vitamin-A, 9-cis-retinoid acid (9-cis), all-trans retinoid acid (ATRA) were from Sigma and solved in sunflower seed oil. Dithizone were from Sigma. The concentration of antibodies used in this study was based on the company instruction.

### Mice and human tissues samples

4–6-week-old male or female C57BL/6 and BALB/c mice were from Beijing Animal Center. TLR2 deficient (−/−) mice and Ob/Ob mice were from Nanjing Animal Center (Nanjing, China). NF-κB deficient (−/−) mice were offered by Prof. Zhexong Lian in Chinese Technique University (Anhui, China). These mice were bred and maintained under specific pathogen-free conditions at the animal facility of Nankai University (Tianjin, China). Germ-free (GF) mice were generated by the Fourth Military Medical University, bred and maintained at the animal facility of the Fourth Military Medical University. Experiments were carried out using age and gender matched groups. All procedures were conducted according to the Institutional Animal Care and Use Committee of the Model Animal Research Center. Animal experiments were approved by the Institute’s Animal Ethics Committee of Nankai University. The experiments for human tissue samples were approved by the Institute’s Ethics Committee of Nankai University and conducted according to the Institutional Use Committee of the Human Tissue Samples. The colon tissues from healthy individuals (gut disease-free) and patients with Crohn’s disease were offered by Tianjin People Hospital. These experiments were approved by all subjects.

### Experimental mice

For microbiota transplantation mice, caecal contents were pooled from five *WT* mice. Each caecal content (150 mg) was sampled in an anaerobic chamber and suspended in 1 mL PBS (phosphate buffered saline) and intragastrically administered (0.1 mL per mouse) immediately to sterilely-packed 6–7 week old germ-free mice three times per week for 4 weeks. *Lacbacillus* NK colony was selected and cultured in *Lactobacillus* selected medium (Barebio., China) and then suspended in PBS and intragastrically administered (1 × 10^9^ CFU/mouse) immediately to sterilely-packed 6–7 week old mice three time per week for four weeks.

For antibiotics-treated mice, 6- to 8-week-old mice were treated with ampicillin (A, 1 g/L, Sigma), vancomycine (V, 0.5g/L), neomycin sulfate (N, 1 g/L), and metronidazole (M, 1g/L) in drinking water for 4 weeks^23^ via the drinking water. Water containing antibiotic was exchanged every three days. To confirm the elimination of bacteria, stool was collected from antibiotic-treated and untreated mice and cultured in anaerobic and aerobic condition. The bacteria were counted under microscope.

For the deletion of Paneth cells, mice were treated with dithizone (100 mg/kg, i.v.)^24^; whereas control mice were treated with Li2CO3 vehicle. Dithizone is dissolved as 10 mg/mL of final concentration in the saturated Li2CO3 (1 g/100 mL). Gut tissues and sera were collected respectively in 0 hrs, 6 hrs, 12 hrs and 6 days for further experiments.

For high-fat-diet mouse model, 6–8 week old mice were fed using high-fat diet (lipid, 18%, yolk powder 10%, cane sugar (10%), cholesterol 1%, bile salts (0.2%) and other basic substance (60.8%) for 3 months.

### *In vitro* stimulation

For *in vitro* stimulation, gut epithelial crypts were stimulated using bacteria (crypt cells: bacteria = 1: 100) or 9-cis-retinoid acid (9-cis, 1μM), all-trans retinoid acid (ATRA, 2μM) for 12 hrs, then lysed for immunoblot of FABP2, FABP4, adipsin and adiponectin. To isolate crypts, samples were transferred to 5 mM EDTA in PBS (pH 8), followed by three 1 min shakings by hand, a 15-min incubation at 4 °C, and passage through 70-μm filters (BD Falcon) to collect the flowthrough. Fraction containing intact and isolated crypts were collected by centrifugation at 75 g for 5 min. at 4^o^C and washed with PBS. No adipocytes and macrophages were detected in the collected intestinal crypts.

To identify gut specific *Lacbacillus* colonies, which could mediate the expression of FABP4, adipsin and adiponectin in gut epithelial crypt cells, isolated bacteria colonies from the culture under strictly anaerobic conditions were killed by heating and then used as stimulator to induce the expression of FABP4, adipsin and adiponectin in the gut epithelial crypts. Finally, 18 bacteria colonies with or without stimulation ability were selected and sequenced.

### Gut *Lacbacillus* colonies

For isolation of gut microbiota, the caecal contents from *WT* mice were serially diluted with PBS and seeded onto *Lacbacillus* selected culture plates. After culture under aerobic conditions or strictly anaerobic conditions at 37 °C for 24 hrs, individual colonies were picked up and cultured for an additional 2 or 4 days at 37 °C in *Lacbacillus* selected culture medium. The isolated colonies were collected into stock medium (10% glycerol) and stored at −80 °C. The sequences of the 16S rRNA of the isolated colonies were obtained by cycle sequencing and then were aligned with the 16S rRNA database of GeneBank using BLAST. Each inquiry gave 100 most similar sequence results including different bacterial genera. For each genus, the one bacterial strain with the highest Max Score was selected and its sequence was downloaded. Next, all obtained sequences were aligned by MUSLE and then neighbor-joining method with a bootstrap of 1000 replicates was used to construct the phylogenetic tree.

### Histological and immunostaining

For histological and immunostaining analysis, previously reported protocol^25^ was used in this study. Briefly, tissues were fixed in 10% formalin-buffered saline and embedded in paraffin, 5-μm-thick sections were prepared from embedded tissue and fixed in acetone (−20 °C) for 10 min. After rehydration in PBS for 5 min and further washing in PBS, tissue sections were blocked with 1% (w/v) BSA and 0.2% (w/v) milk powder in PBS. The primary antibody was added and incubated overnight at 4 °C. After PBS washing (three times, 5 min each), tissue was detected with DAB kit or fluorescence labeled second antibody. Notably, intestinal epithelial Paneth cells could be stained using anti-lysozyme antibody; whereas putative intestinal stem cells, which were localized at the crypt base, were stained by anti-LGR5 antibodies. Nuclei were stained by DAPI.

For immune-staining of intestinal epithelial cells, intestinal epithelial cells (Tongwei company, Shanghai, China) were seeded onto coverslips in 24 well dishes and were grown for 24 hrs, and then the cells were stained with different antibody and second fluorescence-conjugated antibody and then counterstained for nucleic acid with DAPI. Nuclei were stained with DAPI (blue). Cells were analyzed with a Zeiss LSM 710 laser-scanning confocal microscope.

### RT-PCR and qRT-PCR

RT-PCR and qRT-PCR were performed using previously reported protocol^25^. Briefly, semi-quantitative reverse transcription-polymerase chain reaction (RT-PCR) and quantitative real-time PCR (qRT-PCR) were performed. Total RNA was extracted from the cells, tissues and organs using TRIzol reagent (Invitrogen Corp). First-strand cDNA was generated from the total RNA using oligo-dT primers and reverse transcriptase (Invitrogen Corp). The PCR products were visualized on 1.0% (wt/vol) agarose gel. Real-time PCR was conducted using QuantiTect SYBR Green PCR Master Mix (Qiagen) and specific primers in an ABI Prism 7000 analyzer (Applied Biosystems). GAPDH mRNA expression was detected using each experimental sample as an endogenous control. The fold changes were calculated using the ∆∆C_t_ method according to the manufacturer’s instructions (Applied Biosystems). All the reactions were run in triplicate. Following primers were used: adiponectin, 5′TGTTGGAATGACAGGAGCTG and 5′CGAATGGGTACATTGGGAAC; adipsin, 5′GCTATCCCAGAATGCCTCGTT and 5′GGTTCCACTTCTTTGTCCTCGTAT; FABP4, 5′ATGATCATCAGCGTAAATGG and 5′GCCTTTCATAACACATTCCA; FABP2, 5′TTGCTGTCCGAGAGGTTTCT and 5′GCTTTGACAAGGCTGGAGAC; Adipocyte enhancer-binding protein 1 (AEBP-1), 5′CACGTTCCTCGCGCCCTTTC and 5′GTTGCACCATGAATCAGGTC; perilipin1(PLIN1), 5′GGGACCTGTGAGTGCTTCC and 5′GTATTGAAGAGCCGGGATCTTTT; FXIIIA, 5′CAGAGAGACTACCAGAGCACC and 5′GCATTGGAGTTATTGGGCGG; CD11b, 5′CTCCATGCATTGACCTCCCC and 5′GGCATTGGTCACAGGCAAGA; GAPDH, 5′TCAACGGCACAGTCAAGG and 5′TACTCAGCACCGGCCTCA.

### Immunoprecipitation and immunoblot

Immunoprecipitation and immunoblot were performed according to the previously reported protocol^25^. Briefly, the cells were lysed with cell-lysis buffer (Cell Signaling Technology), which was supplemented with a protease inhibitor ‘cocktail’ (Calbiochem). The protein concentrations of the extracts were measured using a bicinchoninic acid assay (Pierce). Immunoprecipitation (IP) was performed essentially the same as described by the manufacturer (Thermo Scientific, USA). Cell lysates were preabsorbed with protein A/G agarose beads at 4 °C for 1 h. After centrifuging, the supernatants were incubated with antibody overnight at 4^o^C followed by incubation with protein A/G agarose beads (Santa Cruz biotechnology) for 2 hr at 4 °C. For the immunoblot, hybridizations with primary antibodies were conducted for 1 h at room temperature in blocking buffer. The protein-antibody complexes were detected using peroxidase-conjugated secondary antibodies (Boehringer Mannheim) and enhanced chemiluminescence (Amersham).

### Serum FABP4, adipsine and adiponecin

Commercial kits for FABP4 (CY-8077, CycLex), adipsine (KA3822, Abnova) and adiponectin (A05187, bertinpharma) were used to quantitate mouse serum FABP4, adipsin and adiponecin. The absorbance on a microplate reader (Labsystems Dragon Wellscan MK2) were detected at a wavelength of 450 nm. The serum levels of FABP4, adipsin and adiponecin were quantified from two to three titrations using standard curves.

### Serum Glu, CHO and TG

Serum glucose (Glu), cholesterin (Cho) and triglyceride (TG) were analyzed using blood biochemical Analyzer (GRT-3006).

### Data analysis

The statistical significance of the comparisons between the two groups was determined using a student’s t-test. The statistical significance of the comparisons between the multiple groups was determined using an ANOVA test. Serum FABP4, adipsin and adiponectin and serum glucose (Glu), cholesterin (Cho) and triglyceride (TG) were analyzed by a Mann-Whitney U test. A 95% confidence interval was considered significant and was defined as P < 0.05. *p < 0.05, **p < 0.01, ***p < 0.001.

## Additional Information

**How to cite this article**: Su, X. *et al.* Expression of FABP4, adipsin and adiponectin in Paneth cells is modulated by gut *Lactobacillus. Sci. Rep.* **5**, 18588; doi: 10.1038/srep18588 (2015).

## Supplementary Material

Supplementary Information
