# Supplementary material for: Expression of FABP4, adipsin and adiponectin in Paneth cells is modulated by gut Lactobacillus
Source: Sci Rep. 2015 Dec 21;5:18588. doi: 10.1038/srep18588 (PMC4685643; doi:10.1038/srep18588)
Supplement: Supplementary Information [file srep18588-s1.pdf]

Supplementary information

Expression of FABP4, adipsin and adiponectin in Paneth cells is modulated by gut *Lactobacillus*

Xiaomin Su[1]*, Hui Yan[1]*, Yugang Huang[1]*, Huan Yun[1], Benhua Zeng[4], Enlin Wang[1], Yu Liu[1], Yuan Zhang[1], Feifei Liu[1], Yongzhe Che[1], Zhiqian Zhang[2] & Rongcun Yang[1,2,3]

[1]Department of Immunology, Nankai University School of Medicine; [2]State Key Laboratory of Medicinal Chemical Biology; [3] Key Laboratory of Bioactive Materials Ministry of Education, Nankai University, Tianjin, P. R. China; [4] The Fourth Military Medical University, Chongqing, P. R. China.

*These authors contributed equally to this paper.

Correspondence and requests for materials should be addressed to Rongcun Yang. (ryang@nankai.edu.cn).

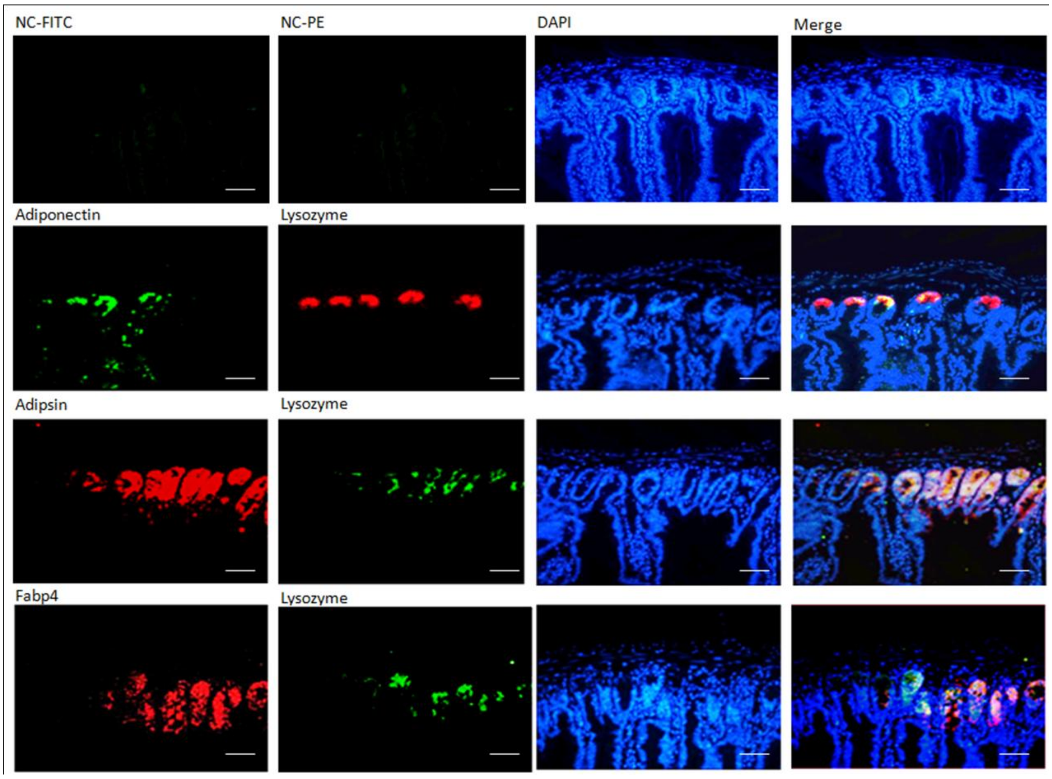

Supplementary Figure S1. Expression of FABP4, adipsin and adiponectin in intestinal Paneth cells. Double immunostaining of FABP4, adipsin and adiponectin with lysozyme in ileum section from *WT* mice. Intestinal tissues were sliced and stained using pooled isotypic controls with FITC-labeled (NC-FITC) or PE-labeled (NC-PE) second antibodies, or anti-lysozyme (lysozyme), anti-FABP4, anti-adipsin or anti-adiponectin followed by fluorescence-labeled second antibodies. Lysozyme (red or green), adiponectin (green), adipsin (red) and FABP4 (red) were shown. Nuclei were stained by DAPI (blue). Scale bar, 40 μm.

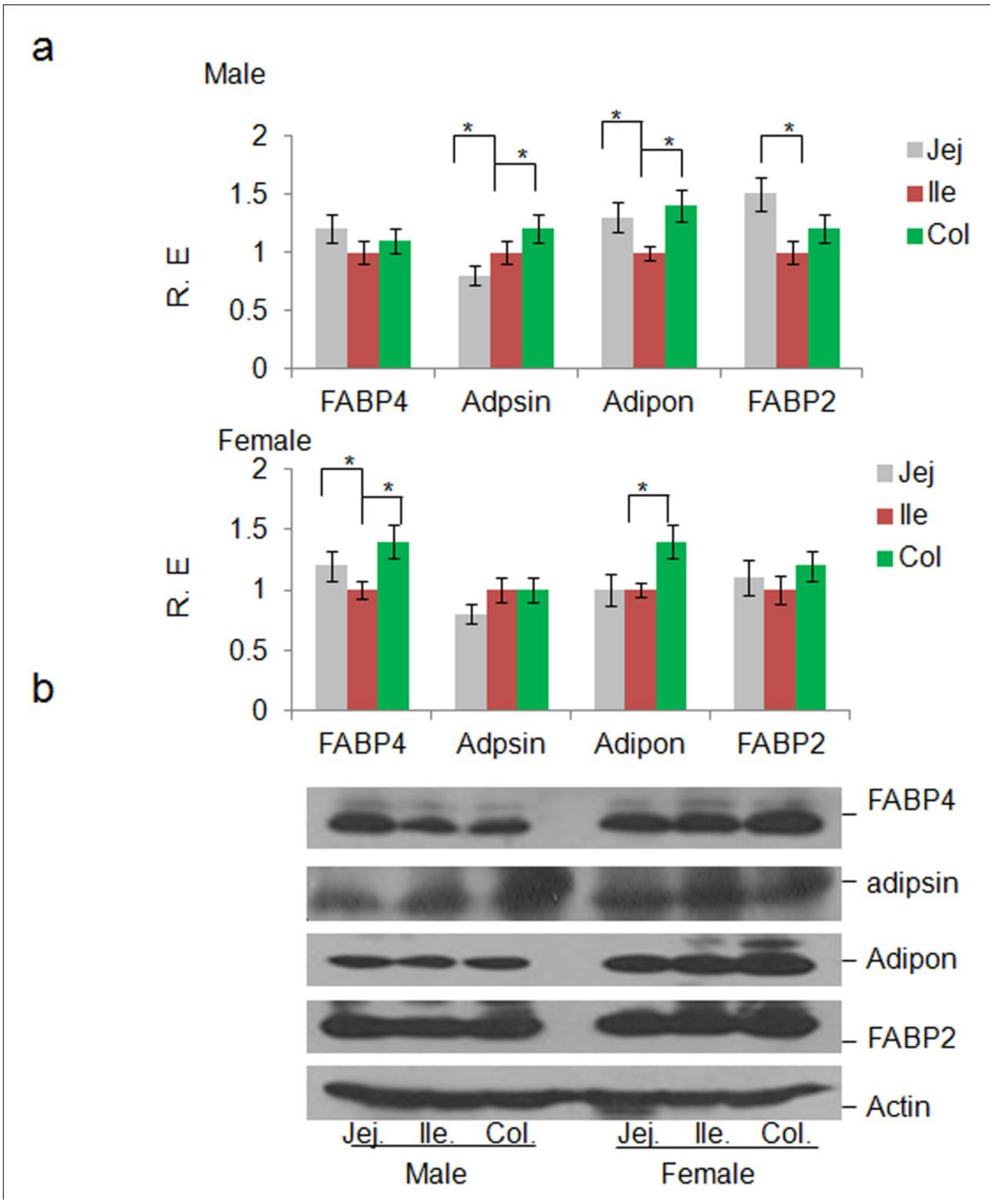

Supplementary Figure S2. Expression of FABP4, adipsin and adiponectin in the epithelial crypt cells of jejunum, ileum and colon of male and female mice. (a) qRT-PCR analyses of FABP4, adipsin, adiponectin (adipon) and FABP2 in the epithelial crypt tissues of jejunum (Jej), ileum (Ile) and colon (Col) of male and female mice (n=6). The transcriptional levels of FABP4, adipsin, adiponectin and FABP2 in ileum fragments were *arbitrarily* designated as 1. R. E., relative expression.

(b) Immunoblot analyses of FABP4, adipsin, adiponectin (adipon) and FABP2 in the epithelial crypt tissues of jejunum (Jej), ileum (Ile) and colon (Col) of male and female mice (n=6). The crypts of jejunum (Jej), ileum (Ile) and colon (Col) tissues of male and female mice were isolated and lysed. The protein levels of FABP4, adipsin, adiponectin and FABP2 were analyzed using immunobot. *P < 0.05 and **P < 0.01 (t-test, mean ± SD). The data are at least a representative of three independent experiments.

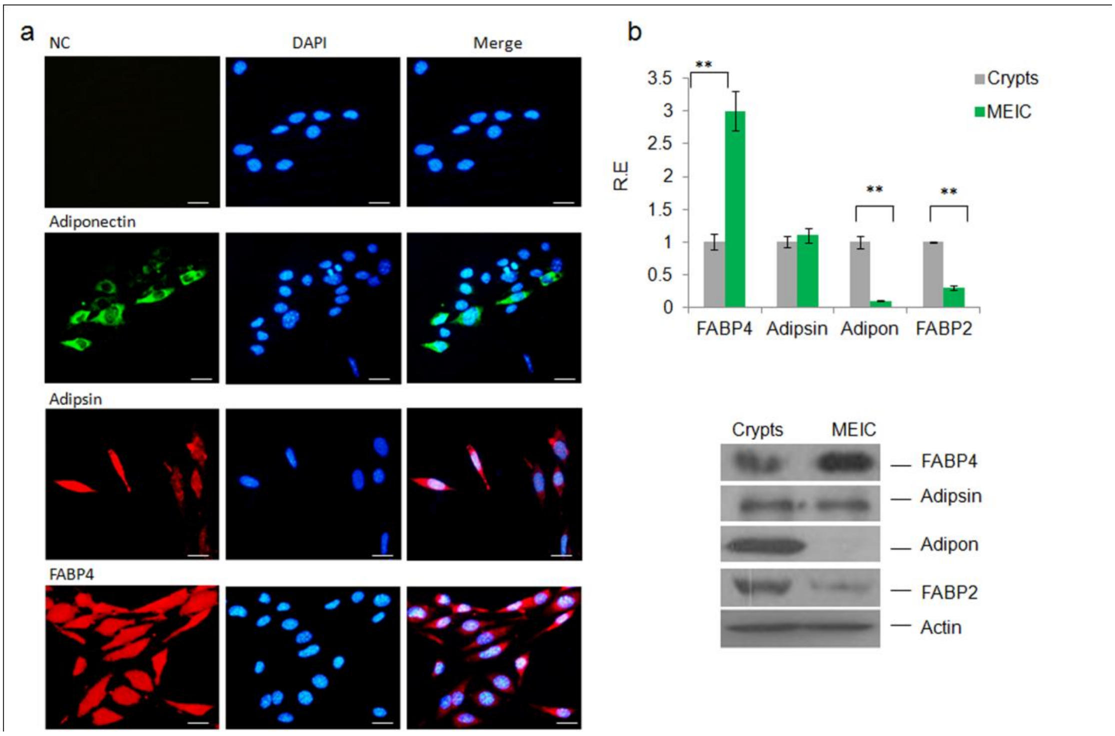

Supplementary Figure S3. Expression of FABP4, adipsin and adiponectin in intestinal epithelial cells. (a) Immunostaining of FABP4 (red), adipsin (red), and adiponectin (green) in mouse intestinal epithelial cells (MIEC) from mouse embryonic intestinal tissues. Scale bars, 40 μm. **(b)** qRT-PCR and immunoblot analyses of intestinal crypts and MIEC. Actin, a loading control, was detected by

anti-β-actin antibody. R. E., relative expression. *P < 0.05 and **P < 0.01 (t-test, mean ± SD). The data were representative of three independent experiments.

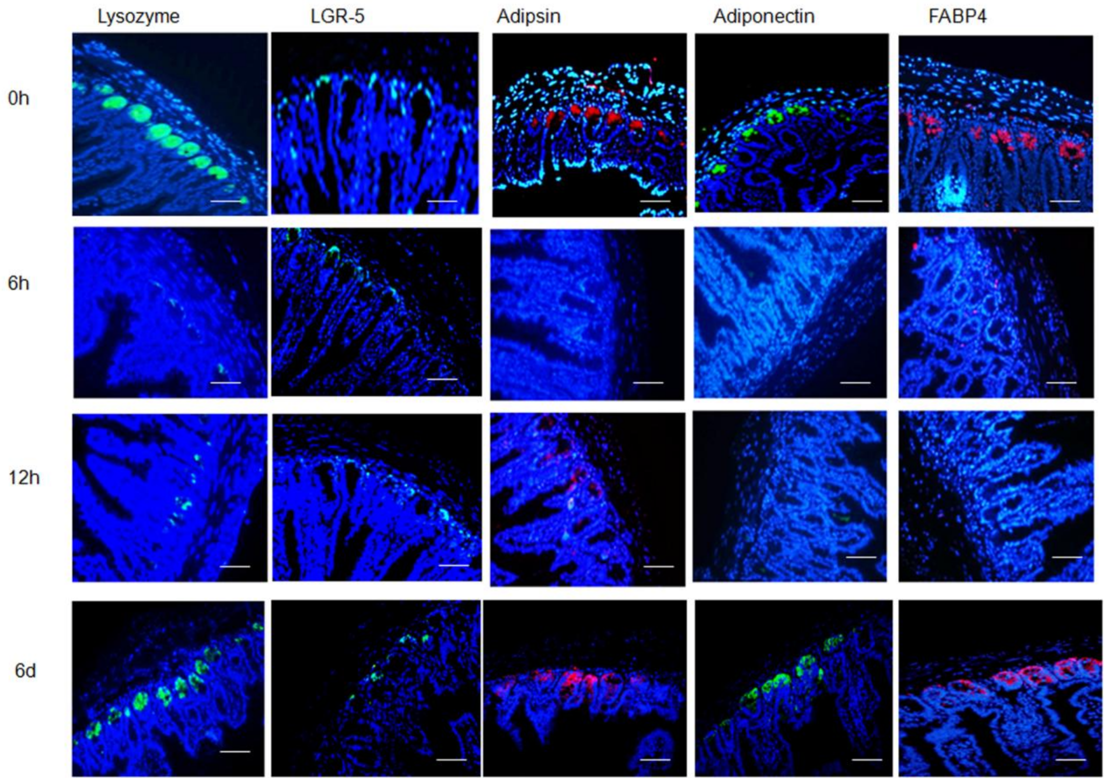

Supplementary Figure S4. Immuno-staining of lysozyme, LGR-5, adipsin, adiponectin and FABP4 in the ileum fragments of mice with and without dithizone treatment. The mice were intravenously injected by dithizone and killed at the indicated time (male, n=6 or indicated number). The ileum fragments from mice with (6 h, 12 h and 6 days after dithizone) and without (0 h) dithizone treatments were sliced and stained by anti-lysozyme, anti-LGR5 or anti-FABP4, anti-adipsin and anti–adiponectin. Red and green indicated respectively FABP4, adipsin or lysozyme, LGR5 and adiponectin. Scale bar, 40 μm.

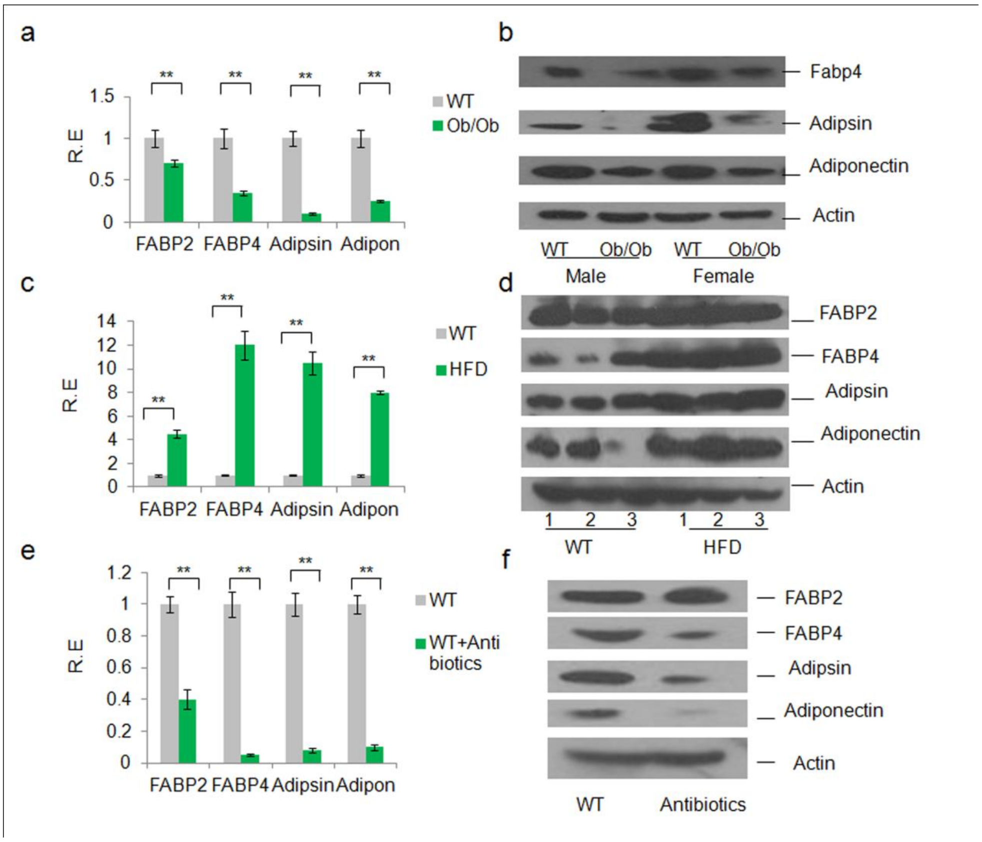

Supplementary Figure S5. Expression of FABP4, adipsin and adiponectin in intestinal epithelial crypt cells of Ob/Ob, high-fat diet and antibiotics-treated mice. (a, c and e) qRT-PCR of FABP2, FABP4, adipsin and adiponectin (Adipon) in ileum fragments of Ob/Ob (a), high-fat diet (c, HFD) and antibiotics-treated (e) mice (n=6). The crypts of ileum fragments of Ob/Ob, HFD and antibiotics-treated mice (antibiotics) were isolated and the transcriptional levels of FABP2, FABP4, adipsin and adiponectin were analyzed using qRT-PCR. R. E., relative expression. (**b**, **d** and **f**) Immunoblot analyses of FABP2, FABP4, adipsin and adiponectin in ileum fragments of Ob/Ob (b), high-fat diet (d) and antibiotics-treated (f) mice. The crypts of ileum fragments of Ob/Ob (n=3), high-fat diet (n=6) and antibiotics-treated mice (n=6) were lysed and the protein levels of FABP2, FABP4, adipsin and adiponectin were

analyzed using immunoblot. *$P < 0.05$ and **$P < 0.01$ (*t*-test, mean ± SD). The data are at least a representative of three independent experiments.

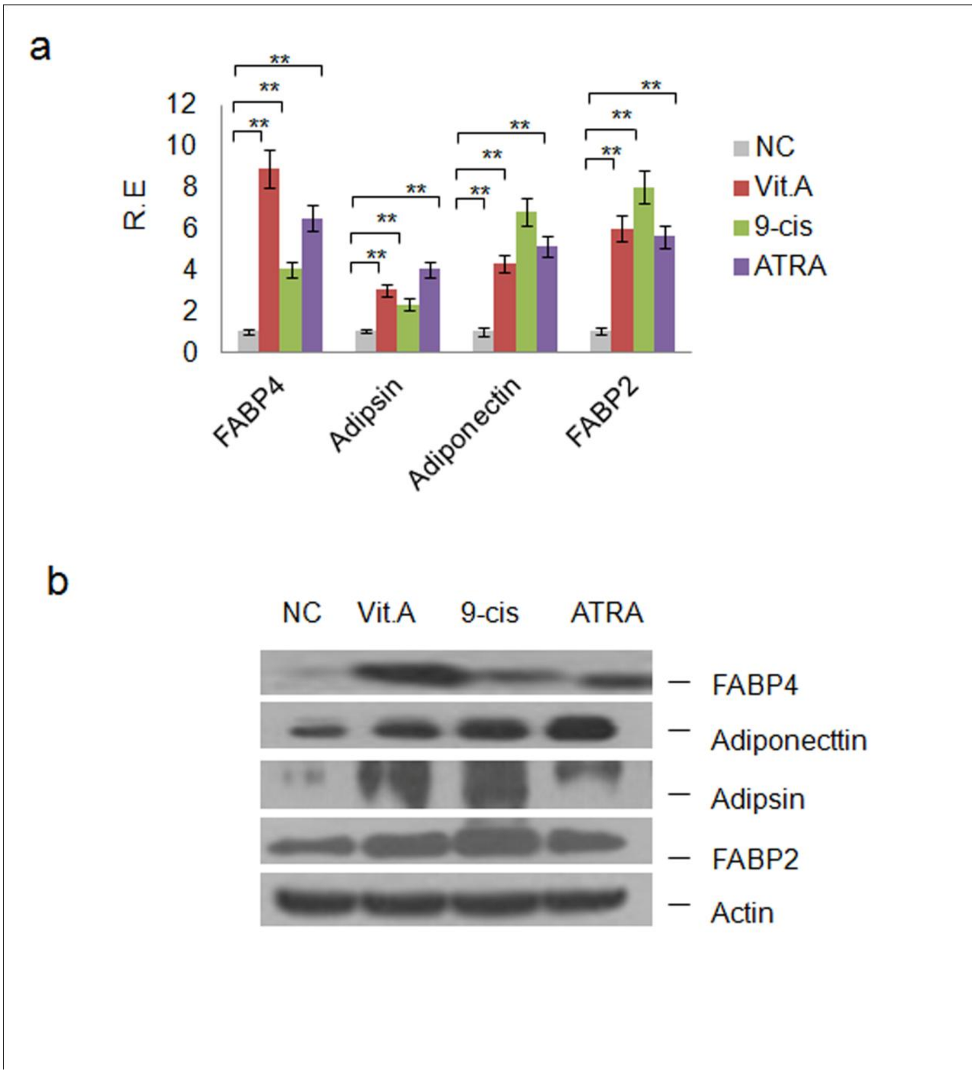

Supplementary Figure S6. Vitamin-A and its metabolites mediate the expression of FABP4, adipsin and adiponectin in gut epithelial crypt cells. (a) qRT-PCR analyses of FABP4, adipsin, adiponecin and FABP2 in the ileum epithelial crypt cells in mice treated with vitamin-A (Vit.A), 9-cis-retinoid acid (9-cis) or all-trans retinoid acid (ATRA). Vitamin-A (14.4μg/mL), 9-cis-retinoid acid (11μg/mL) and all-trans

retinoid acid (33 μg/mL) were solved in sunflower seed oil and then intragastrically administered (100 μl/10g body weight, n=6) immediately to sterilely-packed 6-7 week old mice three time per week for four weeks. NC, negative control; R. E., relative expression. (**b**) Immunoblot analyses of FABP4, adipsin, adiponecin and FABP2 in the ileum epithelial crypt cells of mice treated with vitamin-A (vit. A), 9-cis-retinoid acid (9-cis) or all-trans retinoid acid (ATRA). $^{*}P < 0.05$ and $^{**}P < 0.01$ (t-test, mean ± SD). The data are at least a representative of three independent experiments.

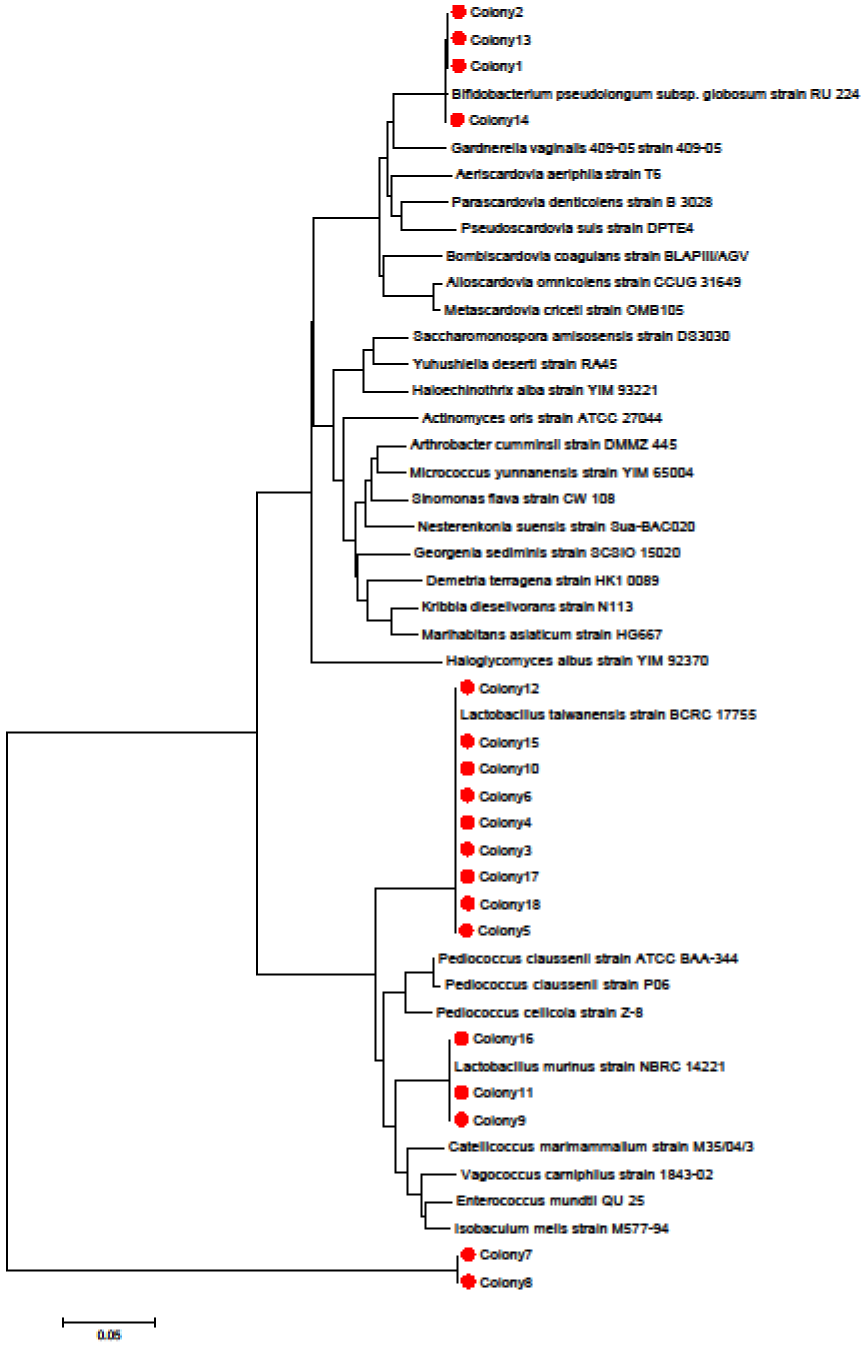
Colony2
Colony13
Colony1
Bifidobacterium pseudolongum subsp. globosum strain RU 224
Colony14
Gardnerella vaginalis 409-05 strain 409-05
Aeriscardovia aeriphila strain T6
Parascardovia denticolens strain B 3028
Pseudoscardovia suis strain DPTE4
Bombiscardovia coagulans strain BLAPIII/AGV
Alloscardovia omnicolens strain CCUG 31649
Metascardovia criceti strain OMB105
Saccharomonospora amisosensis strain DS3030
Yuhushiella deserti strain RA45
Haloechinothrix alba strain YIM 93221
Actinomyces oris strain ATCC 27044
Arthrobacter cumminsii strain DMMZ 445
Micrococcus yunnanensis strain YIM 65004
Sinomonas flava strain CW 108
Nesterenkonia suensis strain Sua-BAC020
Georgenia sediminis strain SCSIO 15020
Demetria terragena strain HK1 0089
Kribbia dieselivorans strain N113
Marihabitans asiaticum strain HG667
Haloglycomyces albus strain YIM 92370
Colony12
Lactobacillus taiwanensis strain BCRC 17755
Colony15
Colony10
Colony6
Colony4
Colony3
Colony17
Colony18
Colony5
Pediococcus claussenii strain ATCC BAA-344
Pediococcus claussenii strain P06
Pediococcus cellicola strain Z-8
Colony16
Lactobacillus murinus strain NBRC 14221
Colony11
Colony9
Catellicoccus marimammalium strain M35/04/3
Vagococcus carniphilus strain 1843-02
Enterococcus mundtii QU 25
Isobaculum melis strain M577-94
Colony7
Colony8
0.05

Supplementary Figure S7. Similarity of *Lacbacillus* NK6 with other bacteria. The gut *Lacbacillus* colonies were selected and cultured in *Lactobacillus* selected medium (Barebio, China). The selected 18 bacteria colonies, which were used in scanning experiments for their ability to induce the expression of FABP4, adipsin and adiponectin, were sequenced. Colonies 6 (*Lacbacillus* NK6) and its similar colonies may effectively mediate the expression of FABP4, adipsin and adiponectin in gut epithelial crypt cells; whereas other clones did not or only induce weak responses (unshown). Most (9 out of 18) of these colonies corresponded to bacteria belonging to *Lactobacillus* genus, which have the highest similarity with *Lactobacillus taiwanensis strain* BCRC 17755. Another 3 *Lactobacillus* colonies are similarity with *murinus strain* NBRC 14221). In addition, 4 colonies represented bacteria of *Bifidobacterium* genus locating at the same branch with *Bifidobacterium pseudolongum subsp globosum strain* RU 224. The bacteria with the greatest p-distance to others were from colony 7 and 8, but the most similar bacterium of them is still *Lactobacillus taiwanensis strain* BCRC 17755 as determined by BLAST.

>A10253_18#_1407035775Y.seq.Contig1TGGATGGACCTGCGGTGCATTAGCTAGTTGGTAAGGTA
ACGGCTTACCAAGGCAATGATGCATAGCCGAGTTGAGAGACTGATCGGCCACATTGGGACTGAGACAC
GGCCCAAACTCCTACGGGAGGCAGCAGTAGGGAATCTTCCACAATGGACGCAAGTCTGATGGAGCAA
CGCCGCGTGAGTGAAGAAGGGTTTCGGCTCGTAAAGCTCTGTTGGTAGTGAAGAAAGATAGAGGTAG
TAACTGGCCTTTATTTGACGGTAATTACCTAGAAAGTCACGGCTAACTACGTGCCAGCAGCCGCGGTAAT
ACGTAGGTGGCAAGCGTTGTCCGGATTTATTGGGCGTAAAGCGAGTGCAGGCGGTTCAATAAGTCTGA
TGTGAAAGCCTTCGGCTCAACCGGAGAATTGCATCAGAAACTGTTGAACTTGAGTGCAGAAGAGGAG
AGTGGAACTCCATGTGTAGCGGTGGAATGCGTAGATATATGGAAGAACACCAGTGGCGAAGGCGGCTC
TCTGGTCTGCAACTGACGCTGAGGCTCGAAAGCATGGGTAGCGAACAGGATTAGATACCCTGGTAGTC
CATGCCGTAAACGATGAGTGCTAAGTGTTGGGAGGTTTCCGCCTCTCAGTGCTGCAGCTAACGCATTAA
GCACTCCGCCTGGGGAGTACGACCGCAAGGTTGAAACTCAAAGGAATTGACGGGGGCCCGCACAAG
CGGTGGAGCATGTGGTTTAATTCGAAGCAACGCGAAGAACCTTACCAGGTCTTGACATCCAGTGCAAA
CCTAAGAGATTAGGTGTTCCCTTCGGGGACGCTGAGACAGGTGGTGCATGGCTGTCGTCAGCTCGTGT
CGTGAGATGTTGGGTTAAGTCCCGCAACGAGCGCAACCCTTGTCATTAGTTGCCATCATTAAGTTGGGC
ACTCTAATGAGACTGCCGGTGACAAACCGGAGGAAGGTGGGGATGACGTCAAGTCATCATGCCCCTTA
TGACCTGGGCTACACACGTGCTACAATGGACGGTACAACGAGAAGCGAACCTGCGAAGGCAAGCGGA
TCTCTTAAAGCCGTTCTCAGTTCGGACTGTAGGCTGCAACTCGCCTACACGAAGCTGGAATCGCTAGTA
ATCGCGGATCAGCACGCCGCGGTGAATACGTTCCCGGGCCTTGTACACACCGCCCGTCACACCATGAG
AGTCTGTAACACCCAAAGCCGGTGGGATAACCTTTATAGGAGTCAG

Supplementary Figure S8. Sequence of *Lacbacillus* NK6. The 16s rRNAs from *Lacbacillus* NK6 were extracted and sequenced by primers (Forward, 5’-AGAGTTTGATCMTGGCTCAG; Reverse, 5’-ACGGCTACCTTGTTACGACTT).

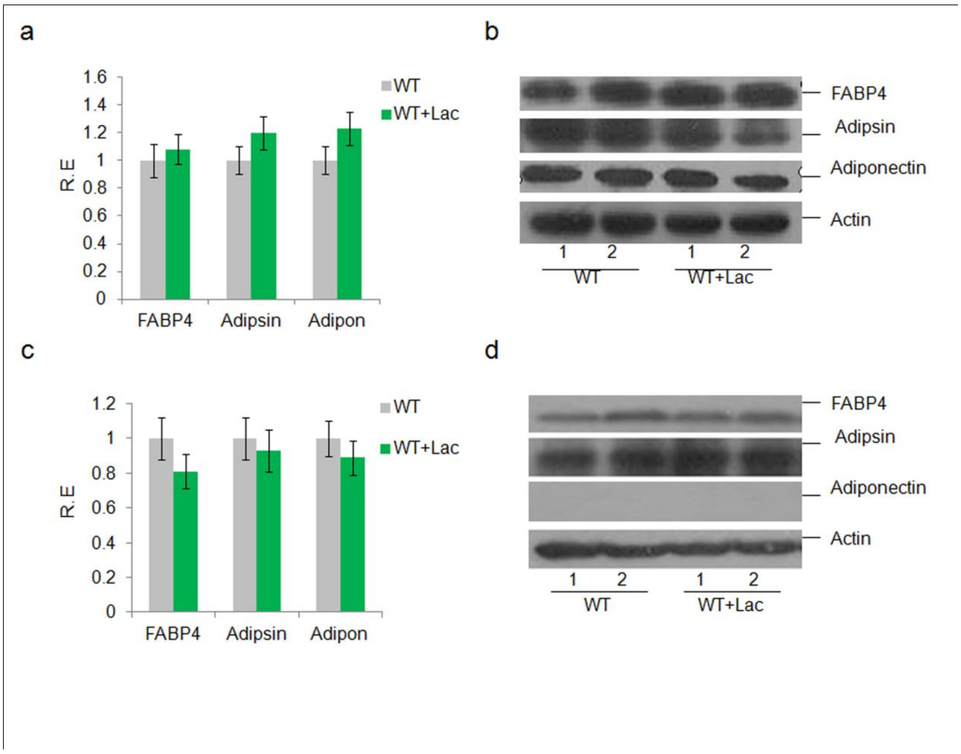

Supplementary Figure S9. Expression of FABP4, adipsin and adiponectin in adipose tissues and macrophages of mice with and without *Lacbacillus* NK6 treatment. (**a** and **c**) qRT-PCR of FABP4, adipsin and adiponectin in adipose tissues (a) and macrophages (c) of mice with (*WT*+Lac, n=6) or without (*WT*, n=6) *Lactobacillus* NK6 treatment. R. E., relative expression. (**b** and **d**) Immunoblot of FABP4, adipsin and adiponectin in adipose tissues (b) and macrophages (d) of mice with (*WT*+Lac., n=6) or without (*WT*, n=6) *Lactobacillus* treatment. Mice were intragastrically administered (1×10^9 per mouse) with *Lactobacillus* NK6 three times per week for 4 weeks. The crypts of ileum fragments were isolated, and the transcriptional and protein levels of FABP4, adipsin and adiponectin were analyzed. Macrophages were harvested from these *Lactobacillus* treated mice by peritoneal lavage 3-4 days following an i.p. injection of 4% sterile thioglycolate medium and then cultured in 6 well plates and incubated at 37°C and 5% CO_2. After 6 hours, the cells were washed to separate out any nonadhesive cells. Macrophage purify reached 93% by FACScan analysis for $CD14^+$ and $CD11b^+$ markers.

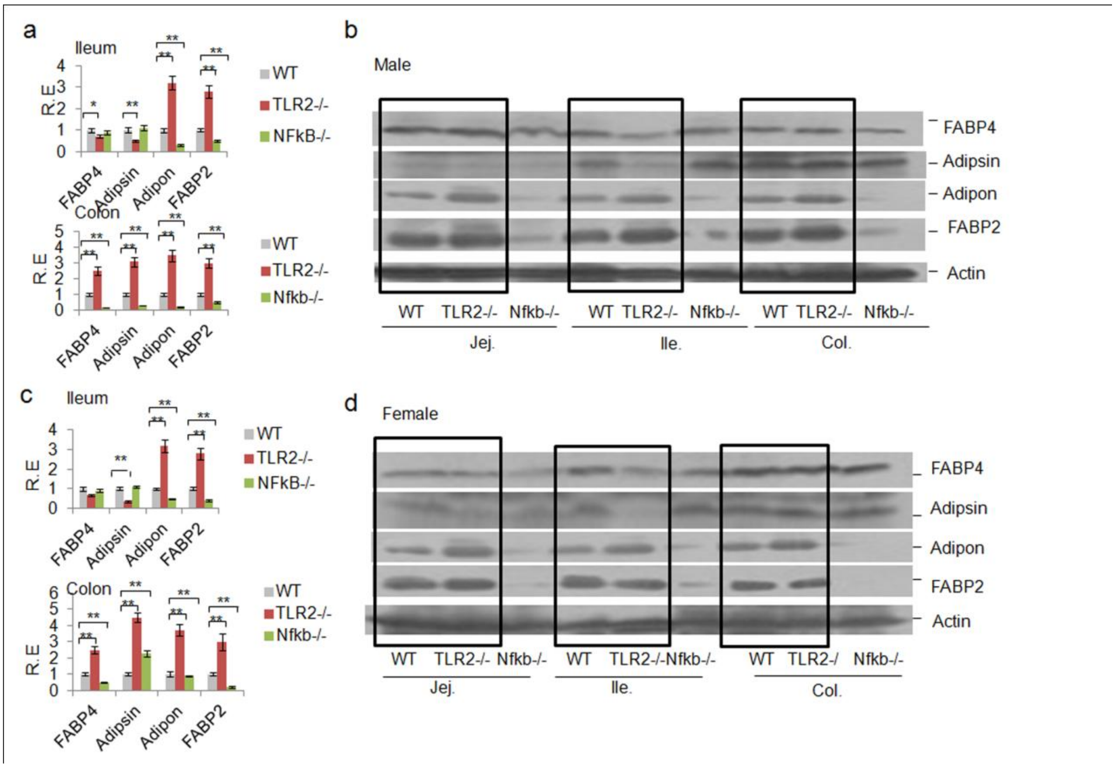

Supplemenatry Figure S10. Expression of FABP4, adipsin and adiponectin in *WT*, TLR2 and NF-κB deficient mice. (a and **c)** qRT-PCR analyses of FABP4, adipsin and adiponecin of intestinal (ileum (Ile)) and colon fragments (Col) in male (a) and female (c) *WT*, TLR2 and NF-κB (Nfkb) deficient (-/-) mice (n=6). The crypt cells of intestinal and colon tissues of TLR2 and NF-κB deficient mice were isolated and the total RNAs were extracted. The transcriptional levels of FABP4, adipsin, adiponectin and FABP2 were detected using qRT-PCR. R. E., relative expression. (**b** and **d**) Immunoblot analyses of FABP4, adipsin and adiponecin of intestinal (jejunm (Jej) and ileum (Ile)) and colon fragments (Col) in male (b) and female (d) *WT*, TLR2 and NF-κB (Nfkb) deficient mice (n=6). The crypts of ileum and colon tissues of TLR2 and NF-κB deficient mice were lysed and the protein levels of FABP4, adipsin, adiponectin and FABP2 were analyzed using immunoblot. *$P < 0.05$ and **$P < 0.01$

(*t*-test, mean ± SD). The data are at least a representative of three independent experiments.

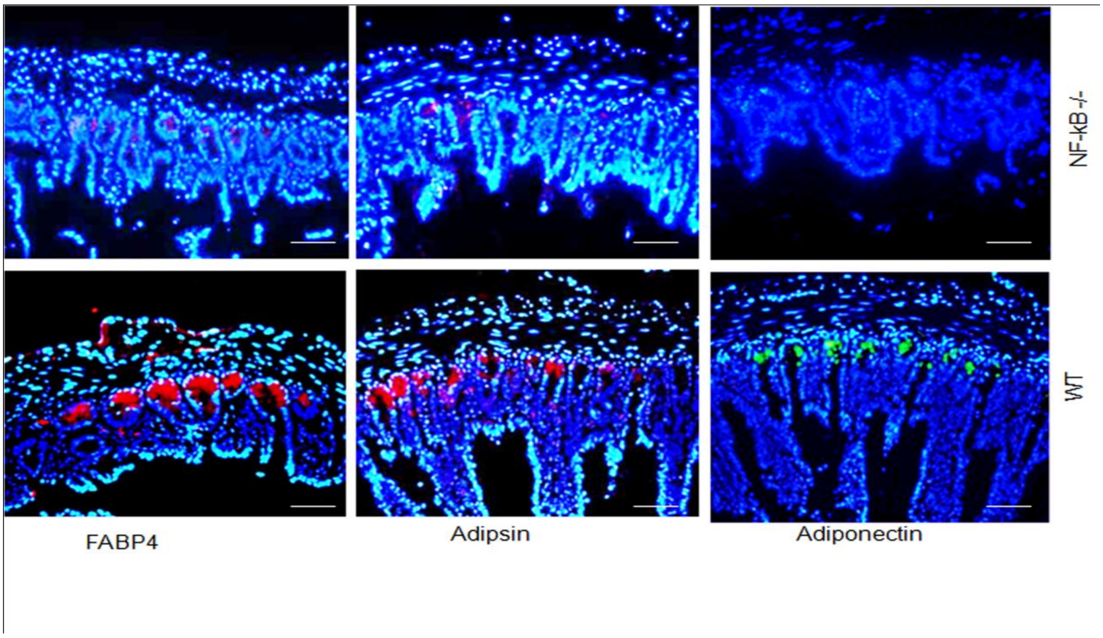

Supplementary Figure S11. Immunohistochemistry analyses of FABP4, adipsin and adiponectin in *WT* and NF-κB deficient mice. Immuno-staining of ileum fragments of *WT* and NF-κB deficient (-/-) mice. The ileum fragments from *WT* or NF-κB deficient mice were sliced and stained by anti-FABP4, anti-adipsin and anti-adiponectin. Red and green indicated respectively FABP4, adipsin or adiponectin. Scale bar, 40 μm. The data are a representative of 6 mice.

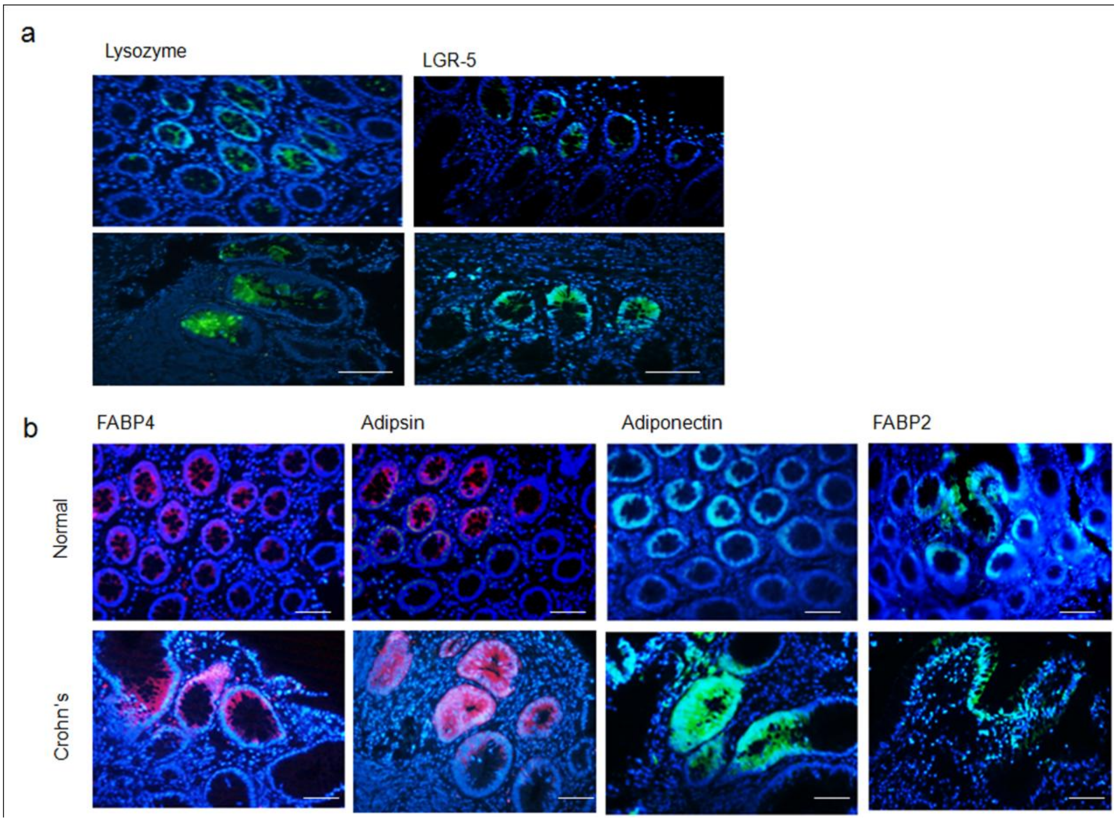

Supplementary Figure S12. Immunohistochemistry analyses of FABP4, adipsin and adiponectin in human colitis epithelial crypt cells. (a) Immuno-staining of lysozyme and LGR5 in colon epithelial cells of healthy individuals and Crohn's disease patients. The colon fragments from healthy individuals (normal) and Crohn's disease (Crohn) were stained by anti-lysozyme or LGR5. Green indicated lysozyme or LGR-5. (**b**) Immuno-staining of FABP4, adipsin and adiponectin in healthy individuals and Crohn's disease patient. The colon fragments from healthy individuals (normal) and Crohn's disease (Crohn) were stained by anti-FABP4, -adipsin and -adiponectin. Red and green indicated respectively FABP4, adipsin and adiponectin or FABP2. Scale bar, 40 μm. The data are a representative of eight cases.
